# The impact of social networks on rural residents’ engagement in living environment upgrade: An integrated analysis drawing on social network theory and the theory of planned behavior

**DOI:** 10.1371/journal.pone.0312779

**Published:** 2025-01-22

**Authors:** Gulsanam Amat, Jing Wang

**Affiliations:** School of Public Administration, Hebei University of Economics and Business, Shijiazhuang, China; Sichuan Agricultural University, CHINA

## Abstract

The issue of rural living environment is a critical concern for China and the global community, intricately intertwined with regional economic development. The proactive involvement of rural residents, who are both beneficiaries and influencers of the environment, constitutes a cornerstone in improving environmental standards. Therefore, the factors that shape rural residents’ participation in living environment upgrade must be explored. Drawing upon the theory of planned behavior and social capital theory, this study employs a structural equation model (SEM) to comprehensively examine the determinants of rural residents’ engagement in living environment upgrade, utilizing data from 334 households in Hebei Province, China. The findings can be summarized as follows: (1) Social networks, subjective norms, perceived behavioral control, and attitudes towards behavior significantly and positively impact rural residents’ engagement in upgrading the living environment, (2) Attitudes towards behavior act as mediators between perceived behavioral control and rural residents’ engagement in living environment upgrade, as well as between subjective norms and rural residents’ engagement in living environmental upgrade. (3) Social networks could indirectly influence rural residents’ engagement in living environment upgrade through the independent mediating effect of subjective norms and attitudes towards behavior, as well as through the chain mediating effect of the two. In conclusion, several managerial recommendations are proposed to stimulate rural residents’ active participation in living environment upgrade.

## Introduction

The environment serves as the fundamental underpinning for human survival and development, necessitating a collective responsibility from the global community to safeguard ecological integrity. According to data provided by the United Nations Population and Development Commission, approximately 44% of the world’s population resides in rural areas as of 2020. In addition, the results of China’s seventh national census reveal that, as of November 2020, 36.11% of the population in China lives in rural areas. In recent years, rapid urbanization has resulted in a continual degradation of environmental quality, posing significant obstacles to rural economic advancement. The contemporary challenge lies in the simultaneous pursuit of sustainable rural living environments and economic development in rural areas.

The quality of the rural living environment in China is profoundly influenced by three major factors: household waste, domestic sewage, and toilet hygiene. These factors impose substantial constraints on the development of rural areas. To comprehensively enhance the quality of the rural living environment, Chinese authorities have successively issued key policies such as the ‘Three-Year Action Plan for Rural Living Environment Improvement’ in 2018 and the ‘Five-year action plan on improving rural living environment (2021–2025)’ in 2021. This series of policies has underscored the necessity of toilet transformations, the segregated handling of household waste, and the sanitation of rural communities, while at the same time raising the importance and the rural living environment upgrade urgency to an unprecedented level [1].

As producers of agricultural products and original inhabitants of rural areas, farmers play a dual role in both contributing to rural pollution and benefiting from environmental improvement [2]. Enhancing rural environmental governance and improving the well-being of rural residents are pivotal in the pursuit of an enhanced livelihood for billions of farmers [3]. However, recent efforts and achievements in rural living environment governance have revealed the pervasive existence of the phenomenon known as “government-led actions with passive public observation” and “pollution followed by remediation” [4], signifying a relatively low level of involvement among rural residents in living environment upgrade. Consequently, there is an urgent need to delve into the factors influencing rural residents’ engagement in living environment upgrade bolster their initiatives in engaging with environmental governance, and address the challenges inherent in rural environment upgrade.

In recent years, scholars have conducted extensive research on the rural living environment upgrade, with a focus on specific facets including toilet revolution [5], solid waste management [6], and domestic wastewater treatment [7]. Within the academic discourse, discussions surrounding the influencing factors of rural residents’ engagement in living environment upgrade predominantly revolve around three key dimensions. Firstly, there are considerations of farmers’ individual attributes, such as age [8], environmental awareness [9], and motivations for seeking welfare benefits [10]. Secondly, social factors come into play, encompassing capital endowment [11], institutional incentives and constraints [12], as well as prevailing social norms [13]. Lastly, village-level factors, such as the level of service orientation among village cadres [14] and the adequacy of transportation infrastructure within villages [15] also contribute significantly to shaping rural residents’ participation behaviors in living environment upgrade.

While scholars have extensively investigated the influencing factors shaping rural residents’ involvement in rural living environment upgrade from diverse research perspectives, the existing research exhibited deficiencies in the following three aspects. (1) The impact of the “acquaintance society” characteristic in rural China had been largely overlooked in prior studies examining the antecedent factors of attitudes and behaviors among rural residents. (2) Previous studies predominantly focused on single factors and lack of exploration into the comprehensive effects of multiple factors. (3) Existing research predominantly employed case analysis or theoretical analysis methodologies, with a paucity of empirical studies conducted from the perspective of rural residents’ involvement. Therefore, drawing upon the theory of planned behavior and social network theory, this paper employed a structural equation model (SEM) to comprehensively analyze the determinants influencing rural residents’ engagement in living environment upgrade using data collected from 334 households in Hebei Province, China. By presenting a comprehensive theoretical framework elucidating the factors underlying rural residents’ behavior towards engaging in living environment upgrade, we aimed to provide a reference basis for formulating strategies to enhance rural living environments in China and other developing countries.

This study contributed to the existing literature in three aspects. Firstly, this study advanced our understanding of the cognitive antecedents affecting the living environment improvement behavior of Chinese rural residents. In particular, we considered the unique characteristics of China’s rural ‘acquaintance society’ and endeavored to present a comprehensive causal chain that encompasses both social contextual factors and individual cognitive factors. Second, we have innovatively developed an extended theoretical model of planned behavior, drawing upon insights from Social Network Theory. This approach not only enriched and strengthened the explanatory and predictive capabilities of TPB but also introduced a novel research perspective for investigating pro-environmental behaviors among rural residents. Last, this article provided empirical evidence to substantiate the positive influence of social networks and TPB variables on rural residents’ engagement in upgrading their living environment, while also investigating the mediating and chain mediation effects, thereby elucidating the mechanisms underlying the occurrence of participation behavior at the micro-level.

The structure of this paper is as follows: Section 2 introduces our conceptual framework and hypotheses. Section 3 describes the study area, data collection, and methodology. Section 4 presents the results of data analysis. Finally, section 5 discusses the findings of the analysis and concludes with implications and research limitations.

## Theoretical foundation and research hypotheses

### Theoretical foundation

The Theory of Planned Behavior (TPB) is a behavioral explanation theory proposed by American scholar Ajzen, built upon the Theory of Reasoned Action. TPB asserts that an individual’s engagement in particular behaviors is shaped by their attitudes toward the behavior (AB), subjective norms (SN), and perceived behavioral control (PBC). Generally, individuals exhibiting positive AB, SN, and PBC are more inclined to harbor stronger intentions and inclinations toward executing specific behaviors [16]. As a theoretical scaffold for explicating individual behaviors, TPB has been extensively applied in various studies concerning environmental improvement behaviors. For instance, Zhang et al. [17], Ejigu et al. [18], Xu Z. et al. [19], and Kumar [20] have separately examined attitudes towards waste management, adoption intentions concerning ecological toilets, inclinations towards engaging in environmentally friendly agricultural practices, as well as patterns of waste recycling among rural populations using TPB framework, Numerous such investigations have demonstrated the effectiveness of TPB in analyzing intentions and behaviors associated with participation in environmental governance [21].

Nevertheless, TPB has been criticized for its disproportionate emphasis on “instrumental components” while largely overlooking “emotional components,” leading to challenges in its theoretical explanatory power [22]. To enhance TPB’s capacity to explain behavior, both domestic and international scholars have undertaken theoretical extensions by incorporating various additional factors. For instance, Zhao integrated Environmental Benefits and the Concept of Human-Nature Coordination into TPB framework to examine how environmental values influence consumers’ willingness to participate in agricultural tourism [23]. Similarly, Shalender introduced the variables of Moral Norm and Environmental Concern into TPB to develop a comprehensive model for predicting the adoption intentions of electric vehicles in India [24]. Generally speaking, compared with the original theory, the expanded Theory of Planned Behavior not only possesses stronger explanatory and predictive power but has also become the main direction for the continuous development and application of this theory in behavioral research.

Although TPB incorporates subjective norms to represent social influence within its analytical framework, it remains fundamentally limited by its research perspective, specifically its inadequate analysis of the internalization process of objective social influences [25]. This limitation impairs the theory’s effectiveness in explaining the behaviors of Chinese residents. Additionally, TPB tends to apply subjective norms in a fragmented manner to address complex social influences, which renders it relatively inadequate for uncovering the deep-rooted issues inherent to China context.

An individual constitutes the sum of their social relationships. In Chinese society, which places a strong emphasis on “relationships” and “reasonableness”, the objective social relationship network plays a significant, and often decisive, role in shaping individuals’ thoughts and behavioral tendencies [26, 27]. This influence is particularly pronounced in rural Chinese communities where “relationship networks” are paramount. According to SNT, these networks endow individuals positioned at network nodes with the ability to both “receive” and “exert” influence, serving as platforms for group information exchange. As a result, social networks (SOC) impact individual cognition and attitudes [28] and facilitate collective action through norms of reciprocity established among network members [29]. Moreover, an individual’s degree of identification with their social network group correlates with their susceptibility to the group’s rules, leading them to align their attitudes and behaviors with those of the group [30]. This reflects the process by which objective social influences permeate individual subjective perceptions, demonstrating how social environmental factors interact with individual subjective cognition and attitudes through social relationship networks.

A comprehensive social network can significantly enhance public trust, norms, and social participation, thereby establishing a robust social capital system [31]. Through mechanisms such as consensus formation, relationship building, and cooperative constraints, SOC can facilitate rural residents’ agreement on participating in the governance of their living environments. Consequently, social networks serve as a pivotal variable in addressing and mitigating the deficiencies in socialization within TPB.

In conclusion, to improve the explanatory power of the model regarding the environmental behavior of rural residents in China, this study extends TPB by incorporating SNT into its framework. This integration seeks to address the limitations of SN and AB in explaining environmentally friendly behaviors among rural Chinese residents from an objective social influence perspective. Meanwhile, this is also a manifestation of our commitment to interpreting the behaviors of rural residents in China from the perspective of “relationship orientation” advocated by previous scholars [32].

**Fig 1:**
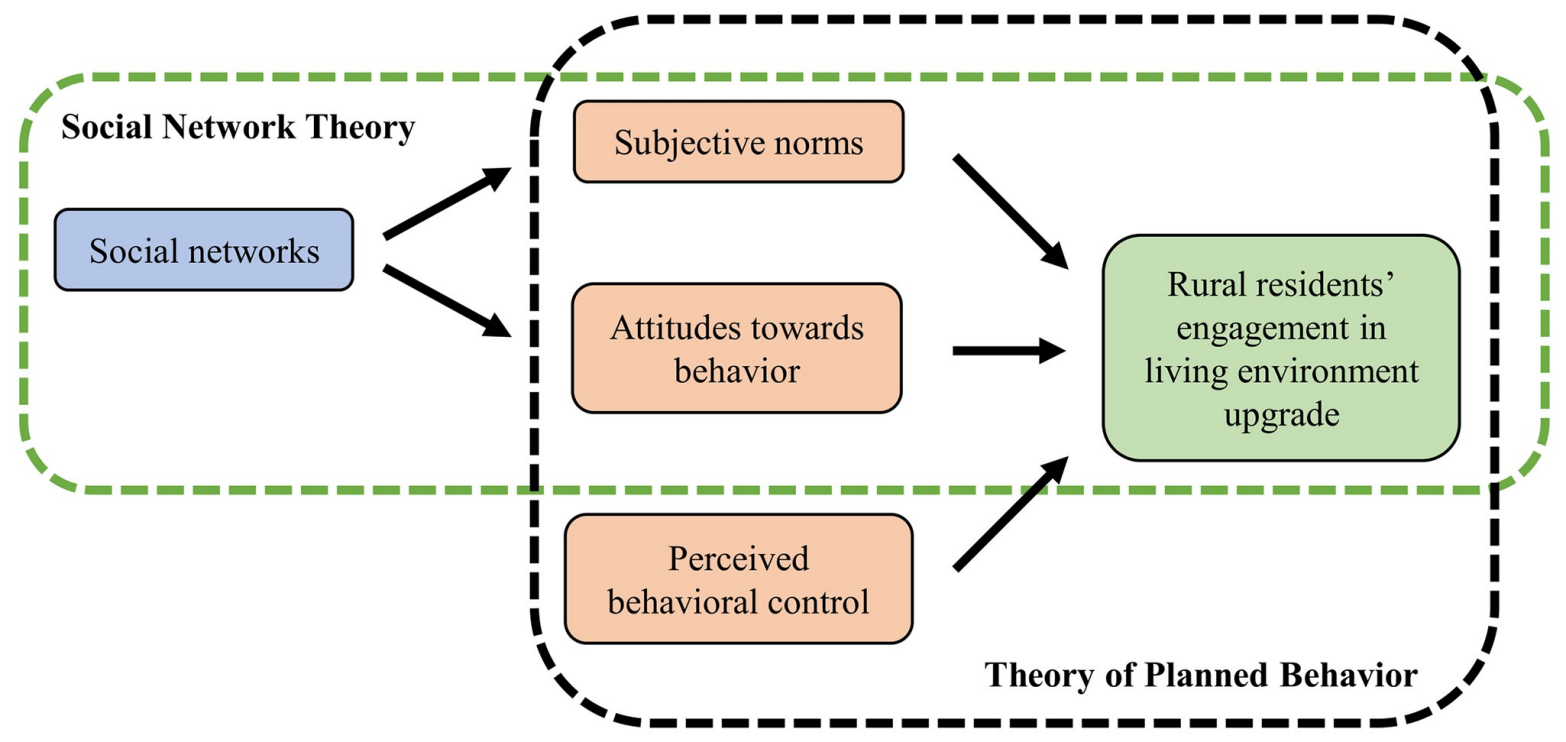
Expanded theoretical framework.

On the basis of the aforementioned description, this paper incorporated the SOC variable, which represents the “emotional components” of individuals, into the theory of planned behavior to establish an extended TPB. Subsequently, the paper employed SEM to undertake empirical analysis on rural residents’ engagement in rural living environment upgrade (ELEU), offering theoretical support for further clarifying the formation process of ELEU. The extended theory of planned behavior model constructed by this paper is presented in Fig 1.

### Research hypotheses

#### Social networks and rural residents’ engagement in living environment upgrade

Social networks represent a structurally stable framework of interpersonal relationships established through ongoing social interactions [33]. Notably, rural China epitomizes a societal structure characterized by familial ties, geographic proximity, and occupational connections [34]. Consequently, the behavioral choices of rural inhabitants are significantly shaped by the intricate web of social networks within which they are situated [35]. Su and Feng’s study elucidates that individuals with broader social networks are more inclined to assume influential roles in social interactions, thus facilitating the expression of their opinions and fostering a stronger inclination towards collective action [36]. The broader an individual’s social network, the greater the opportunities they have to engage in the exchange of information and resources with fellow network members. This exchange catalyzes environmental information spillover, thereby enhancing the individual’s environmental consciousness and nurturing intentions for environmental protection [37]. Academic research has further validated the constructive influence of social networks on rural residents’ engagement in environmental improving endeavors. Recent research highlights the essential role of social networks in advancing the adoption of renewable energy technologies [38], nurturing pro-environmental intentions [39], and fostering engagement in environmental governance [40]. Building upon these empirical insights, we posited the hypothesis as following.

**Hypothesis 1 (H1).** Social networks positively influence rural residents’ engagement in living environment upgrade.

#### Social networks, subjective norms, and rural residents’ engagement in living environment upgrade

Subjective norms denote the social pressures exerted on individuals in making decisions regarding particular behaviors, encompassing the influence of others’ behaviors, and group norms [41]. It is widely acknowledged that individuals with positive subjective norms exhibit stronger behavioral intentions [42]. Research by Razali F has demonstrated a significant correlation between social pressure and individuals’ intentions to correctly dispose of household waste [43].

Social norms are formulated under the sway of social factors [44]. Thus, institutional regulations are inherently constrained by these norms [25]. Professor Lin Nan accentuated that social networks necessitate individuals to ground their rational behaviors on collective action principles and to align their actions with collective interests [45]. In rural China, characterized by dense social networks, credit and reputation serve as forms of “social currency”, and individuals are highly protective of their personal identity and social status, a concept commonly referred to as “face-saving”. Consequently, they conform to the expected behaviors associated with their network roles [46], lest they face social condemnation. It can be posited that social networks furnish the essential assurances for collective action by imposing potential behavioral constraints and internal institutional norms on individuals [47]. This phenomenon is also recognized as the social network regulatory mechanism [48]. Given this foundation, we posited the following hypotheses.

**Hypothesis 2a (H2a).** Social networks positively influence subjective norms.
**Hypothesis 2b (H2b).** Subjective norms positively influence rural residents’ engagement in living environment upgrade.
**Hypothesis 2 (H2).** Subjective norms mediate the relationship between social networks and rural residents’ engagement in living environment upgrade.

#### Social networks, attitudes towards behavior, and rural residents’ engagement in living environment upgrade

Attitudes towards behavior refer to an individual’s subjective assessment of their preference for engaging in a particular behavior. It is generally acknowledged that individuals with a favorable attitude towards behavior demonstrate a greater propensity for making behavioral decisions [41]. Gerald confirmed the role of individuals’ attitudes towards behavior in predicting their environmental managerial behaviors [49].

China’s rural communities are commonly described as “acquaintance societies” [41] or “semi-acquaintance societies” [50], wherein individuals cultivate social relationships characterized by reciprocal influence [51]. These relationships directly or indirectly influence the consciousness and behavior of villagers. Bott’s research underscores the pivotal role of the relational structure within social networks in shaping and reshaping individual perceptions [52]. As previously indicated, social networks play a pivotal role in the dissemination of information, and it is noteworthy that these networks are generally built upon a foundation of trust. For rural residents, information shared within their social networks carries greater credibility than that from third-party sources [53]. Information regarding the benefits and significance of enhancing living conditions, disseminated through social networks [54], can effectively reshape villagers’ attitudes towards environmental improvements and inspire their adoption of environmentally friendly behaviors [55]. Attitudes serve as a crucial mediating factor in this process. Gilletta has also emphasized that individuals’ attitudes and behaviors in specific issues are becoming more alike because of dynamic opinion formation and information sharing within social networks [35]. Based on the aforementioned discussion, we proposed the following hypotheses.

**Hypothesis 3a (H3a).** Social networks positively influence attitudes towards behavior.
**Hypothesis 3b (H3b).** Attitudes towards behavior positively influence rural residents’ engagement in living environment upgrade.
**Hypothesis 3 (H3).** Attitudes towards behavior mediate the relationship between social networks and rural residents’ engagement in living environment upgrade.

#### Perceived behavioral control, attitudes towards behavior, and rural residents’ engagement in living environment upgrade

Perceived behavioral control refers to an individual’s subjective assessment of his ownability and perceived difficulty in performing a specific behavior [56]. It can be categorized into internal and external dimensions. Internal perceived behavioral control encompasses personal attributes that individuals possess when executing behaviors, such as skills, time, and economic conditions. External perceived behavioral control pertains to the perceived ease or difficulty associated with implementing behaviors, including convenience factors, opportunities, and external challenges for implementation [57]. According to TPB, a stronger perceived behavioral control towards a particular activity is positively correlated with a more positive inclination to engage in it [41]. Numerous scholars have conducted empirical investigations to examine the predictive role of perceived behavioral control on individual behavior, For instance. Parveen’s research revealed that individuals’ perception of behavioral control can influence their efforts to mitigate urban air pollution [58]. Similarly, Lili D highlighted that perceived behavioral control positively influences Chinese residents’ willingness to adopt desalinated water [59]. Furthermore, Li Y’s study discovered that rural resident’s economic income and governance subsidies are significant explanatory factors for their decision-making regarding participation in toilet revolution initiatives [60].

TPB also posits that individuals’ attitudes towards behavior are significantly influenced by their perceived behavioral control [41]. Specifically, when residents perceive themselves as having sufficient ability, convenience, and positive social support to participate in improving their living environment, their attitudes towards such behavior tend to be more favorable. Prior research has demonstrated that villagers’ behavioral decisions can be indirectly influenced by perceived behavioral control through its impact on their attitudes towards behavior [61]. Expanding on this foundation, we posited the following hypotheses.

**Hypothesis 4a (H4a).** Perceived behavioral control positively influences attitudes towards behavior.
**Hypothesis 4b (H4b).** Perceived behavioral control positively influences rural residents’ engagement in living environment upgrade.
**Hypothesis 4 (H4).** Attitudes towards behavior mediate the relationship between perceived behavioral control and rural residents’ engagement in living environment upgrade.

#### Subjective norms, attitudes towards behavior, and rural residents’ engagement in living environment upgrade

The Cognitive Dissonance Theory suggests that individuals consciously adjust their behavioral attitudes to conform to collective norms or align with influential figures within a group [62], indicating a positive impact of subjective norms on behavioral attitudes. Empirical evidence further supports this notion. For instance, Arundati’s analysis demonstrates the positive influence of subjective norms on consumers environmental behavior attitudes [63], while Budovska argues for a significant positive correlation between subjective norms and individuals’ pro-environmental attitudes [64]. Furthermore, empirical studies by Chang [65] and Ajzen [66] have revealed that the inclusion of the “subjective norms-attitudes towards behavior” path in the initial TPB model resulted in a significant improvement in the model’s goodness-of-fit, Therefore, we also suggest that attitudes towards behavior may mediate the relationship between subjective norms and behavioral intentions.

Finally, this paper aimed to construct a comprehensive theoretical framework by integrating subjective norms and attitudes towards behavior as the intermediary factors in a chain mediation model. Specifically, the shaping of behavioral attitudes relies heavily on subjective norms [67]. On the one hand, attitudes exhibit social attributes. Individuals within social networks consciously adjust their viewpoints to align with other members of the group and foster group coordination [62]. For instance, the participatory behavior of members within social networks shapes the attitudes and intentions towards participation of other members [68]. On the other hand, subjective norms, serving as the value orientation of the group, represent a crucial avenue for individuals to access external information [10]. Information obtained from the group enhances their comprehension of the relevant values and potential benefits associated with engaging in the improvement of the living environment, thereby reinforcing their positive attitudes. Essentially, as subjective norms strengthen, villagers’ attitudes towards participation tend to become more proactive and affirmative. Therefore, we proposed the following hypotheses:

**Hypothesis 5a (H5a).** Subjective norms positively influence attitudes towards behavior.
**Hypothesis 5 (H5).** Attitudes towards behavior mediate the relationship between subjective norms and rural residents’ engagement in living environment upgrade.
**Hypothesis 6 (H6).** Subjective norms and attitudes towards behavior play a chain mediation role between social networks and rural residents’ engagement in living environment upgrade.

**Fig 2:**
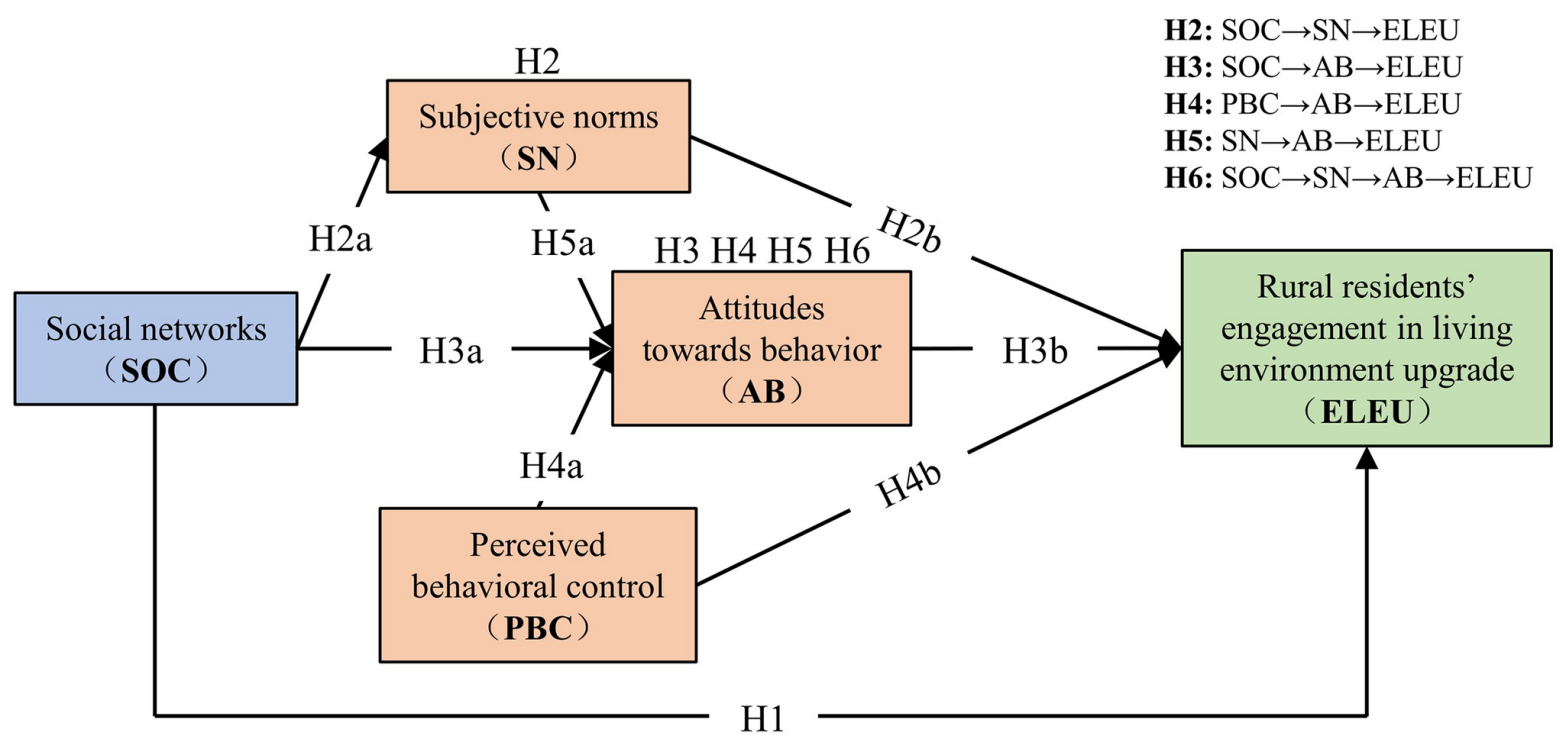
The hypotheses model.

In summary, the hypothetical model constructed was shown in Fig 2.

## Data and methods

### Data sources and sample characteristics

#### Case selection and study area

The research data utilized in this study was garnered from a comprehensive survey focusing on rural revitalization efforts, spearheaded by our research team within Hebei Province, China, spanning the period from December 2021 to January 2022. Geographically, Hebei Province is positioned within the North China Plain, encompassing coordinates ranging from 36°05′ N to 42°40′ N and from 113°27′ E to 119°50′ E. The topography of Hebei Province highlights elevated terrain in the northwest and lower terrain in the southeast, presenting a diverse geographical landscape. According to the results of China’s seventh national census as of November 2020, the permanent resident population of Hebei Province was estimated to be approximately 74.61 million, positioning it as the sixth most populous province across the country. Notably, 39.93% of this population resides in rural areas.

Throughout the past century, Hebei’s steel industry has experienced a remarkable growth, fueled by its abundant resources, advanced technological capabilities, and advantageous proximity to the capital city. Nevertheless, alongside the rapid economic expansion, the environmental landscape in Hebei Province witnessed a persistent decline in quality. These environmental challenges have posed significant obstacles to industrial progress within the province, prompting a pivot towards prioritizing environmental remediation efforts.

**Fig 3:**
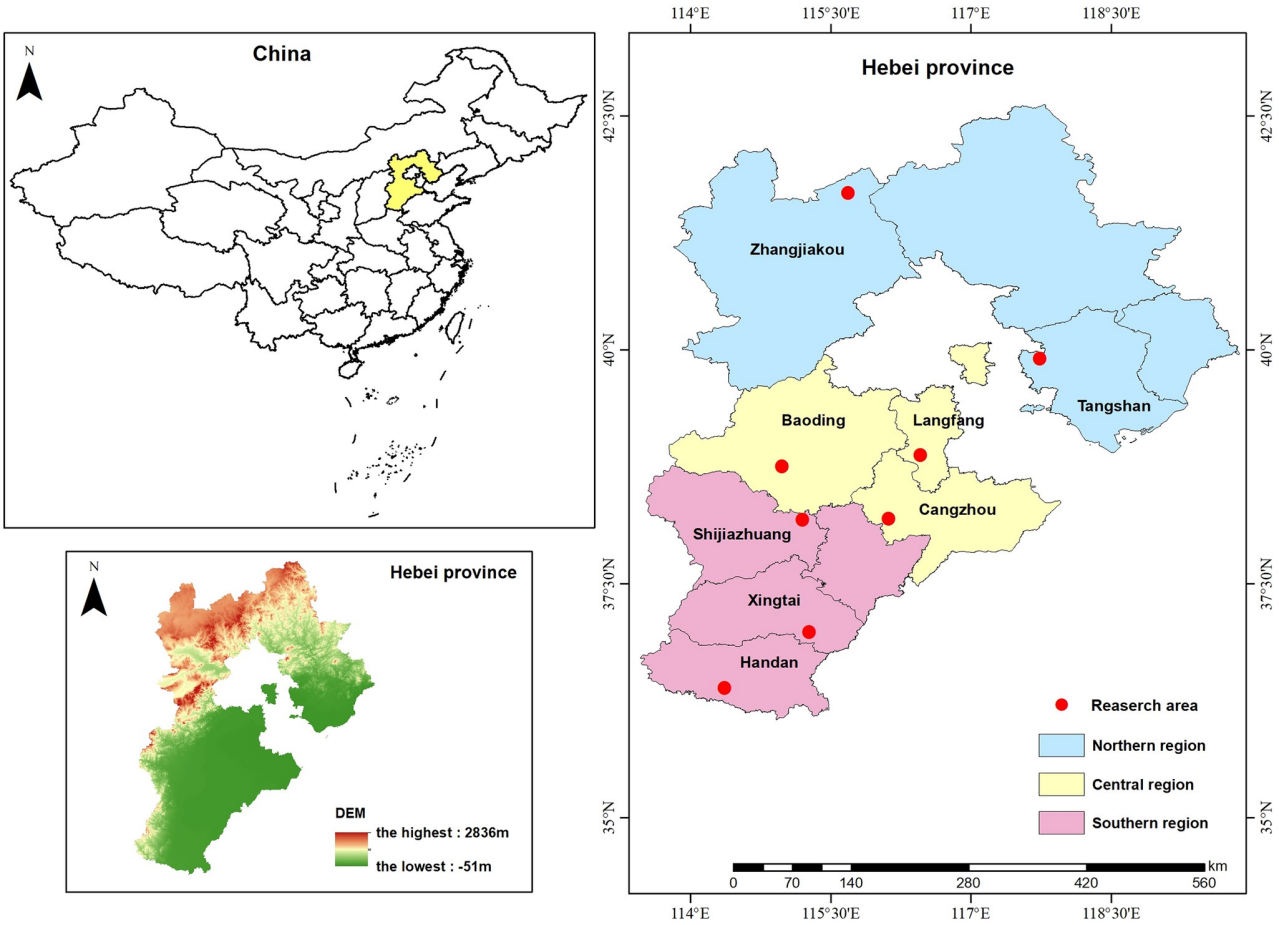
Research area.

The rural regions of Hebei Province sprawl extensively and harbor dense populations, accentuating the intricacies of environmental governance. The establishment of sustainable and thriving villages holds paramount importance for the province. Following years of dedicated environmental improvement endeavors, notable strides have been achieved in improving the rural living conditions in Hebei, garnering recognition from governmental bodies and the populace alike. Hence, we selected Hebei Province as our research site due to its significant reference value and representativeness. Fig 3 delineated the surveyed area.

#### Study design and data collection

Our research team comprised teachers and graduate students with extensive experience in field surveys, a profound understanding of rural life, and thorough training. The research sample was evenly distributed across the northern, central, and southern areas of Hebei Province. Specifically, the northern region includes Zhangjiakou and Tangshan; the central region encompasses Langfang, Cangzhou, and Baoding; while the southern region covers Shijiazhuang, Xingtai, and Handan (as depicted in Fig 3). In total, 8counties, 43 villages, and 341 households were involved in this survey. The survey covered basic information about family households in villages, as well as aspects related to rural industry, ecological environment, culture, services, and livelihoods. This paper primarily focused on the “ecological environment” related questions in the questionnaire. The field survey method yielded a total of 341 household questionnaires, from which 334 valid responses were obtained after excluding invalid ones. The Harman one-way test was employed to assess homophily bias in the data. Analysis of the unrotated factors of all questionnaire items revealed that the first principal component explained 28.701% of the total variance, which was below the threshold of 40%. This indicated that homophily bias was not observed in the data.

#### Sample characteristics

The sample statistical results of this study were presented in Table 1. The surveyed households exhibited the following characteristics: a higher proportion of males compared to females. Most rural residents were aged 60 and above, while those aged 35 and below had the lowest proportion. Among rural residents, the education level was mainly junior high school and below, with agriculture being their primary occupation. Most individuals were married, and the largest proportion consisted of families with 1–2 members. Additionally, most villages were situated within a distance of less than 5km from townships. Furthermore, many villages conducted competitions on household hygiene and have been bestowed with the title of civilized village.

**Table 1:** Descriptive statistics of the sample.

Item	Category	Frequency	Percentage
Gender	Male	218	65.3
	Female	116	34.7
Age	35 and below	17	5.1
	36–59	154	46.1
	60 and above	163	48.8
Nationality	Han nationality	307	91.9
	Minority nationalities	27	8.1
Occupation	Farming	186	55.7
	Non-farming	148	44.3
Educationlevel	Illiterate	44	13.2
	Primary school	109	32.6
	Junior high school	133	39.8
	Middle high school/Technical secondary school	45	13.5
	Junior college/Undergraduate college	3	0.9
Everservedasavillageofficial	No	270	80.8
	Yes	64	19.2
Maritalstatus	Married	307	91.9
	Single/Widowed/Divorced	27	8.1
Numberoffamilymembers	1–2	116	34.7
	3–4	100	29.9
	5–6	84	25.1
	7–8	32	9.6
	9 and above	2	0.6
Distancefromvillagetotown	5 km and below	231	69.2
	6 km and above	103	30.8
Sanitationassessmentsarerequired	Yes	220	65.9
	No	114	34.1
Everawardedthetitleofcivilizedvillage	Yes	134	40.1
	No	200	59.9
Sampledistribution	Northern region	80	24
	Central region	129	38.6
	Southern region	125	37.4
Total		334	100

### Variable measurements

#### Dependent variable: Rural residents’ engagement in living environment upgrade (ELEU)

The “Five-Year Action Plan for Improving Rural Human Settlement Environment (2021–2025),” issued by the Chinese government in 2021, emphasized five critical areas: the toilet revolution, domestic wastewater treatment, domestic waste management, enhancement of village aesthetics, and the establishment of long-term maintenance mechanisms. Among these, the first four aspects constitute the primary construction tasks aimed at improving rural living environments. Building on the findings of relevant studies [69, 70], we have identified and selected four representative, highly effective, and feasible measures for enhancing rural living environments. These measures form the core elements of our proposed improvement strategy. Specifically, this paper employed four questions—“Have you carried out or planned to carry out a harmless sanitary toilet renovation or construction at your home?” “Do you discharge your household wastewater into the public sewer system or into your own sewage facilities?” “Do you utilize the village’s garbage collection facilities?” and “Do you cultivate trees or flowers within your own courtyard?”—as indicators representing rural residents’ engagement in living environment governance. Subsequently, we assigned values to the responses of rural residents regarding their engagement in these four participation behaviors as follows: “l = participated in 0 items; 2 = participated in 1 item; 3 = participated in 2 items; 4 = participated in 3 items; and 5 = participated in 4 items”. The score reflected the level of participation in improving the living environment, with higher scores correlating to greater levels of participation.

Table 2 displayed the participation rates of residents in four environmental improvement initiatives in Hebei Province. It is evident that residents in Hebei Province exhibited the highest enthusiasm for solid waste management, with a participation rate of 92%. This was followed by domestic wastewater treatment, which had a participation rate of 61%. The lowest rates of participation were found in toilet revolution and village appearance improvement, both with a participation rate of 46%.

**Table 2:** Description of rural residents’ engagement in living environment upgrade.

Item	Question	Measure	Mean	SD
Toilet revolution	Have you carried out or planned to carry out a harmless sanitary toilet renovation or construction at your home?	1 = Yes;0 = No	0.46	0.499
Domesticsewagetreatment	Do you discharge your household wastewater into the public sewer system or into your own sewage facilities?	1 = Yes;0 = No	0.61	0.488
Solidwastemanagement	Do you utilize the village’s garbage collection facilities?	1 = Yes;0 = No	0.92	0.278
Villageappearanceimprovement	Do you engage in tree planting or flower cultivation within your own courtyard?	1 = Yes;0 = No	0.46	0.499

#### Explanatory variables

(1) Social networks (SOC). The measurement of social networks includes assessing their scope and intensity [71]. We defined scope using personal networks [72] and intensity through neighborhood ties [73] and official-mass relationships [74].
(2) Perceived behavioral control (PBC). Perceived behavioral control can be measured via internal perceived behavioral control and external perceived behavioral control [58]. In this paper, internal perceived behavioral control among rural residents was evaluated based on their self-evaluations of economic conditions [75], available resources, and time [76], while the availability of external assistance [77] was used to measure external perceived behavioral control.
(3) Subjective norms (SN). Subjective norms can be assessed through subjective beliefs and motivation to comply [78]. In this study, subjective beliefs were measured using “attitudes endorsed by trusted individuals” [79], while motivation to comply was gauged by “support for village regulations” [80] and the “overall community atmosphere” [81].
(4) Attitudes towards behavior (AB). Attitudes towards behavior can be measured by the behavioral beliefs and the outcome evaluations [16]. We measured “behavioral beliefs” through the attitude towards payment [82], while “outcome evaluation” was gauged based on improvements in quality of life [10, 61] and village cleanliness [83].

In summary, each latent variable was operationalized by three measurement items. To ensure the scale’s content validity, all measurement items were adapted from the existing literature. The above specific items and their sources are shown in Table 3. All items were measured using a five-level scale.

**Table 3:** Variables and measurement questionnaire survey.

Latent Variables	Observed Variables	Items	Measure	Measure	Supporting References
ELEU	Rural Residents’ Engagement in Living Environment Upgrade	ELEU	How many of the following four rural living environment improvement items have you participated in: toilet revolution, domestic sewage treatment, domestic waste management, and village appearance enhancement?	1 = Participated in 0 items; 2 = Participated in 1 item; 3 = Participated in 2 items; 4 = Participated in 3 items; 5 = Participated in 4 items	[69, 70]
SOC	Network Range	SOC1	For celebratory events held at your residence, such as weddings, funerals, birthdays, etc., what is the anticipated number of guests you plan to invite?	1 = Less than 50; 2 = 50–99; 3 = 100–149; 4 = 150–199; 5 = 200 or more	[72]
	Network Intensity	SOC2	How many neighbors do you estimate would be willing to provide financial assistance in the event of a family member’s urgent need for 5000 RMB due to illness?	1 = Negligible or absent; 2 = Approximately 20%; 3 = Around 50%; 4 = Roughly 80%; 5 = All	[73]
		SOC3	How would you describe your relationship with village cadres?	1 = Very distant; 2 = Quite distant; 3 = Average; 4 = Quite close; 5 = Very close	[74]
PBC	Internal PBC	PBC1	How would you classify the current economic status of your family within this village?	1 = Lowest level; 2 = Second lowest level; 3 = Average level; 4 = Second highest level; 5 = Highest level	[75]
		PBC2	possess sufficient resources and time to engage in living environment upgrade.	1 = Strongly disagree; 2 = Disagree to some extent; 3 = Neutral; 4 = Agree to some extent; 5 = Strongly agree	[76]
	External PBC	PBC3	It is easy for me to get help from my neighbors.	Same as above	[77]
SN	Subjective Beliefs	SN1	The people I trust believe that participating in the living environment upgrade is worthwhile.	1 = Strongly disagree; 2 = Disagree to some extent; 3 = Neutral; 4 = Agree to some extent; 5 = Strongly agree	[79]
	Motivation to Comply	SN2	The village regulation expects me to participate in the living environment upgrade.	Same as above	[80]
		SN3	The overall atmosphere of the village encourages me to participate in the living environment upgrade.	Same as above	[81]
AB	Behavioral Beliefs	AB1	I think it’s worth paying for managing affairs related to our living environment, like building eco-friendly toilets, installing sewer pipes, and so on.	1 = Strongly disagree; 2 = Disagree to some extent; 3 = Neutral; 4 = Agree to some extent; 5 = Strongly agree	[82]
	Outcome Evaluations	AB2	I think that participating in the living environment upgrade will make my life more convenient.	Same as above	[10, 61]
		AB3	I think that my participation in the living environment upgrade will contribute to enhancing the cleanliness standards in the village.	Same as above	[83]

### Method

This study aimed to examine the following pathway mechanisms:

(1) The direct impact of social networks(SOC), attitudes towards behavior(AB), subjective norms(SN), and perceived behavioral control (PBC) on rural residents’ engagement in living environment upgrade (ELEU).
(2) The mediating and chain-mediating roles of attitudes towards behavior (AB) and subjective norms SN within the extended TPB regarding rural residents’ engagement in the upgrading of living environment.

To achieve the research objectives outlined above, this study employed SEM for a comprehensive analysis. SEM offers advantages over traditional regression models by allowing simultaneous analysis of multiple independent and dependent variables while assessing both direct and indirect effects. The research sample was analyzed using SPSS 27.0 for descriptive statistics, reliability analysis, validity analysis, and correlation analysis. AMOS 24.0 was utilized for conducting structural equation modeling analysis, while PROCESS 4.1 was employed to explore mediating and chain mediation effects.

## Results

### Model fitness test

#### Reliability test

We used Cronbach’s a to test the reliability, and the results are summarized in Table 4. The value of the Cronbach’s a of the four constructs were 0.752, 0.756, 0.787, 0.741, respectively, which were all greater than 0.7, suggesting that the data reliability met the criteria for subsequent analysis [84].

**Table 4:** Reliability and convergent validity test.

Variable	Code	Factor Loading	AVE	CR	Cronbach’s *α*	KMO	Sig
SOC	SOC1	0.693	0.5137	0.7582	0.752	0.684	0.000
	SOC2	0.811					
	SOC3	0.635					
PBC	PBC1	0.725	0.5103	0.7576	0.756	0.694	0.000
	PBC2	0.712					
	PBC3	0.706					
SN	SN1	0.710	0.585	0.8033	0.787	0.635	0.000
	SN2	0.942					
	SN3	0.603					
AB	AB1	0.744	0.5064	0.7538	0.741	0.68	0.000
	AB2	0.750					
	AB3	0.635					

#### Validity test

We conducted Kaiser-Meyer-0lkin (KMO) and Bartlett’s test of sphericity on the four latent variables in our study. The KMO values for all variables exceeded 0.5, and the Bartlett’s test yielded a significance level below 0.001. These results indicated the suitability of the measurement indicators for factor analysis [85, 86].

Subsequently, confirmatory factor analysis (CFA) was conducted to test convergent validity and discriminant validity. We tested the convergent validity by employing standardized factor loadings, composite reliability (CR), and average variance extracted (AVE) of the variables [87]. As displayed in Table 4, the standardized factor loadings for each variable ranged between 0.603 and 0.942, falling within the recommended interval [0.5, 0.95]. This indicated a favorable internal consistency of the measurement model and satisfied the fundamental requirements for analysis [84]. Moreover, both the AVE values and CR values for each latent variable were greater than 0.5 and 0.7 respectively, indicating good convergent validity of the questionnaire and an excellent fit of the model.

As suggested by Fornell and Larcker [87], the discriminant validity of the measurement model distinction was deemed acceptable when the square root of the AVE for each construct exceeded the correlation coefficient between the latent variable and other variables. In this model, the square root of AVE on the diagonal of Table 5 was much greater than the non-diagonal values. Therefore, the discriminant validity between the latent variables in this model was good.

**Table 5:** Discriminate validity test.

Variable	Mean	SD	SOC	PBC	SN	AB
SOC	3.591	0.872	**0.752**			
PBC	3.327	0.784	0.239	**0.756**		
SN	2.92	0.867	0.26	0.237	**0.787**	
AB	3.179	0.741	0.367	0.271	0.277	**0.741**

Note: Values (bold) on the diagonal represent the square root of the AVE while the off-diagonals are correlations.

#### Path structure of structural equation model

**Fig 4:**
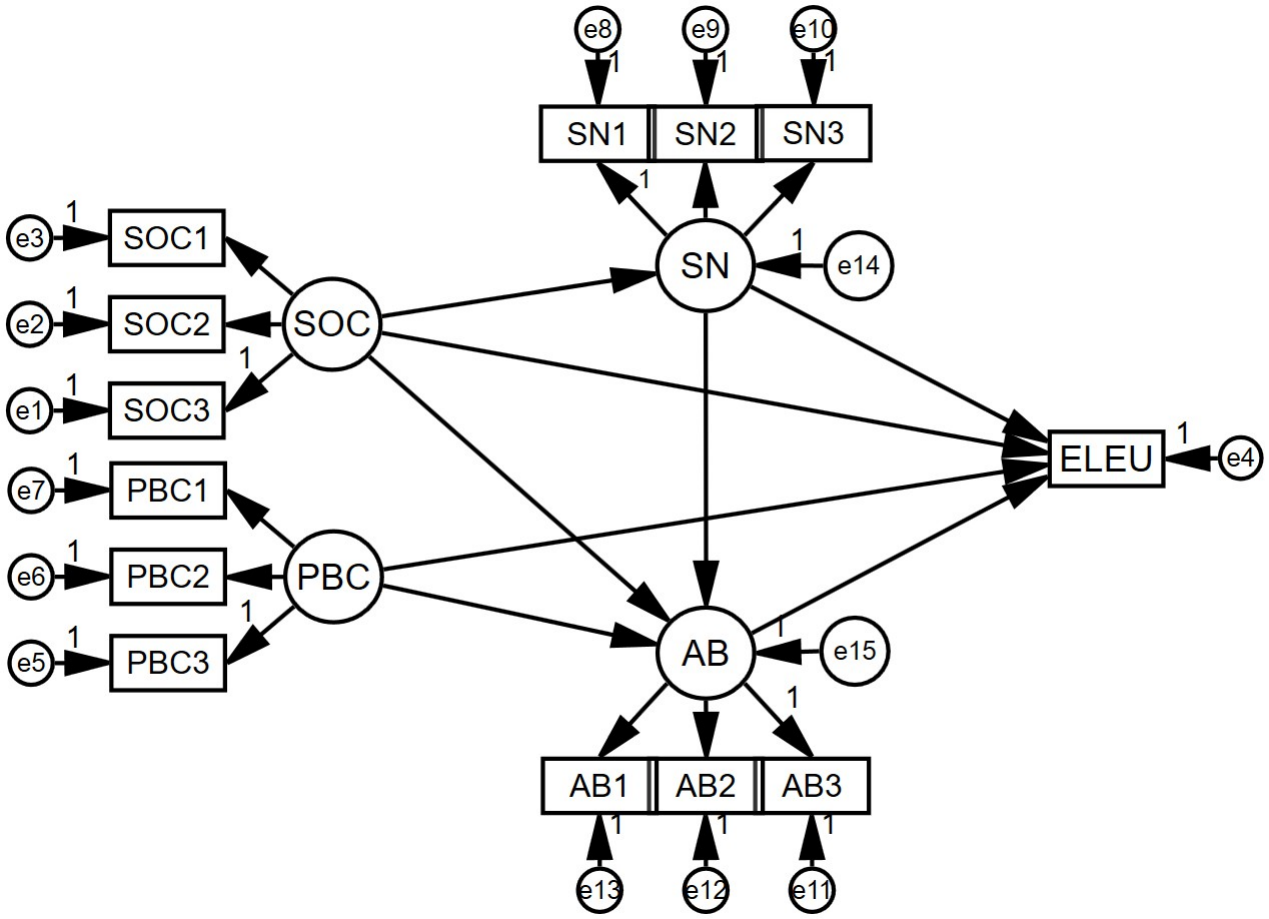
Structural equation model diagram.

After conducting the validity and reliability tests, we utilized Amos 24.0 to construct a structural equation model based on the theoretical framework and hypotheses of our research, as depicted in Fig 4.

#### Goodness-of-fit test

The Goodness-of-Fit test of the constructed model was conducted using a maximum likelihood estimation in AMOS 24.0. The results are presented in Table 6. All estimated values fell within an acceptable range, indicating a favorable fitness of the model [88].

**Table 6:** Model fitness test.

Index	X^2^/df	RMSEA	GFI	AGFI	NFI	CFI	IFI	PNFI	PCFI	PGFI
Recommended Value	< 3.00	< 0.08	> 0.90	> 0.90	> 0.80	> 0.80	> 0.80	> 0.50	> 0.50	> 0.50
Estimated Value	2.402	0.065	0.939	0.905	0.896	0.936	0.937	0.666	0.696	0.599

### Hypotheses test

#### Direct effect test

**Fig 5:**
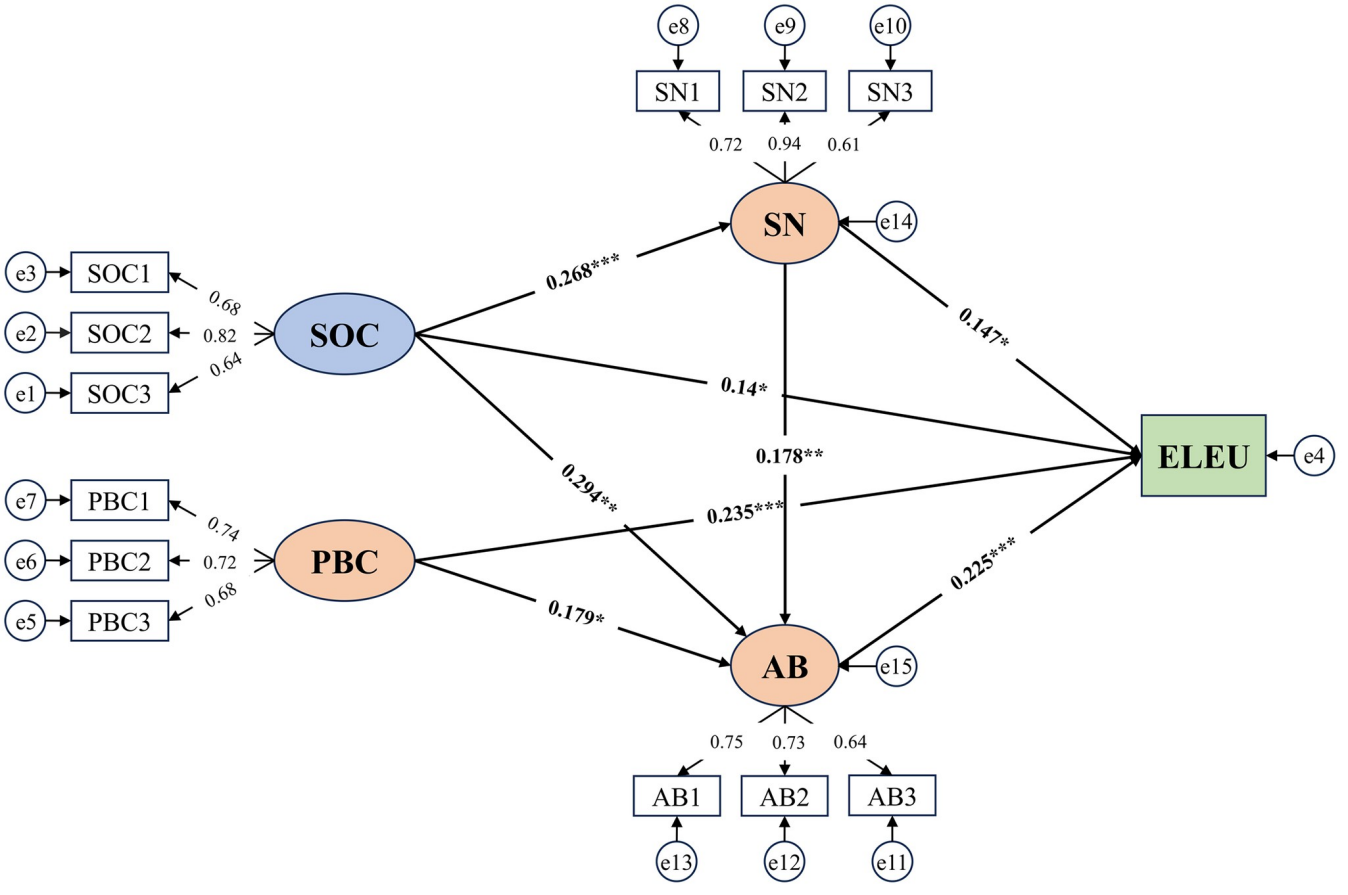
Parameter path of the structural equation model standardized path coefficient estimates. (* means p<0.05, ** means p<0.01, *** means p<0.001).

Table 7 shows the results of the direct effects of the hypothesized model with path coefficients in a standardized form, while Fig 5 shows the parameter paths of the hypotheses in the model. As depicted in Table 7, we have substantiated that the four explanatory variables significantly and positively influenced ELEU, thus providing support for Hl, H2b, H3b, and H4b. Moreover, SOC were found to have a significant positive influence on SN (*β*=0.268, p<0.001), indicating support for H2a. Furthermore, our findings demonstrated a significant influence of SOC (*β*=0.294, p<0.01), PBC (*β*=0.179, p<0.05), and SN (*β*=0.178, p<0.01) on AB, therefore, H3a, H4a, and H5a were supported.

**Table 7:** Results of direct effects.

Path	Estimate	S.E.	C.R.	P	Hypotheses	Result
SOC→ELEU	0.14	0.085	2.198	0.028	H1	Supported
SOC→SN	0.268	0.062	3.893	0.000	H2a	Supported
SN→ELEU	0.147	0.084	2.579	0.01	H2b	Supported
SOC→AB	0.294	0.07	3.918	0.008	H3a	Supported
AB→ELEU	0.225	0.096	3.333	0.000	H3b	Supported
PBC→AB	0.179	0.073	2.559	0.01	H4a	Supported
PBC→ELEU	0.235	0.09	3.871	0.000	H4b	Supported
SN→AB	0.178	0.07	2.638	0.008	H5a	Supported

#### Mediation effect test

To test the mediation hypotheses of social norms (H2) and attitudes towards behavior (H3, H4, and H5), we used PROCESS 4.1 to calculate the 95% confidence intervals (CI) of indirect effects based on 5,000 times bootstrapping [89]. As shown in Table 8, the results demonstrated that SN mediated the relationship between SOC and ELEU (indirect effect = 0.073, 95%CI=[0.031,0.125]). Likewise, the following indirect paths were supported: SOC→AB→ELEU (indirect effect = 0.095, 95%CI=[0.046,0.154]), PBC→AB→ELEU (indirect effect = 0.078, 95%CI=[0.032,0.133]), SN→AB→ELEU (indirect effect = 0.077, 95%CI=[0.034,0.13]). Thus, the four hypotheses regarding mediation effects (H2, H3, H4, H5) were all supported.

**Table 8:** Results of mediation effects.

Path	Type of Effect	Effect	Boot SE	Boot LCI	Boot UCI	Effectiveness Ratio	Hypotheses	Result
SOC→SN→ELEU	Total effect	0.296	0.062	0.175	0.417	100%	H2	Supported
	Direct effect	0.223	0.061	0.104	0.342	75.34%		
	Indirect effect	0.073	0.024	0.031	0.125	24.66%		
SOC→AB→ELEU	Total effect	0.296	0.062	0.175	0.417	100%	H3	Supported
	Direct effect	0.201	0.062	0.079	0.322	67.91%		
	Indirect effect	0.095	0.027	0.046	0.154	32.09%		
PBC→AB→ELEU	Total effect	0.392	0.067	0.26	0.524	100%	H4	Supported
	Direct effect	0.314	0.066	0.184	0.449	80.1%		
	Indirect effect	0.078	0.026	0.032	0.133	19.9%		
SN→AB→ELEU	Total effect	0.379	0.061	0.26	0.498	100%	H5	Supported
	Direct effect	0.302	0.06	0.184	0.42	79.68%		
	Indirect effect	0.077	0.024	0.034	0.13	20.32%		

#### Chain mediation effect test

Additionally, we performed further analysis using the Bootstrap method to assess the chain mediation effect of subjective norms and attitudes towards behavior. The results of this analysis are presented in Table 9. The results demonstrated that SOC had a direct effect on ELEU (*β*=0.154, 95%CI=[0.035,0.274]). Moreover, the total indirect effect was 0.142 which contained three paths: SOC affected ELEU through SN (*β*=0.062, 95%CI=[0.023,0.11]); SOC affected ELEU through AB (*β*=0.068, 95%CI=[0.028,0.12]); and SOC affected ELEU through the chain of SN and AB (*β*=0.012, 95%CI=[0.003,0.25]). The confidence interval of each path did not contain 0, indicating that the significant direct effect of social networks on rural residents’ engagement in living environment upgrade, the significantly mediating effect of subjective norms, attitudes towards behavior, and the chain “subjective norms → attitudes towards behavior” between social networks and the rural residents’ engagement in living environment upgrade have been verified. Hence, the hypothesis H6 was supported.

**Table 9:** Results of the chain mediation effects.

Type of Effect	Effect	Boot SE	Boot LCI	Boot UCI	Effectiveness Ratio	Hypotheses	Result
Total effect	0.296	0.062	0.175	0.417	100%	H6	Supported
Direct effect	0.154	0.061	0.035	0.274	52.03%		
Indirect effect	0.142	0.031	0.085	0.208	47.97%		
Ind1:SOC→SN→ELEU	0.062	0.022	0.023	0.11	20.95%		
Ind2:SOC→AB→ELEU	0.068	0.024	0.028	0.12	22.97%		
Ind3:SOC→SN→AB→ELEU	0.012	0.006	0.003	0.025	4.05%		

## Discussion

This paper explored the factors influencing rural residents’ engagement in upgrading their living environment, drawing on TPB and SNT. We extended the TPB model by integrating social networks as a novel antecedent variable and tested our hypotheses using SEM. The research findings were summarized as follows:

Firstly, our findings demonstrated that social networks effectively facilitate rural residents’ engagement in improving their living environment, supporting the conclusions drawn by Bodin [90]. Specifically, the coefficient of social network observation variables, including social ties, neighborhood relations, and the relationships between cadres and the masses, exceeded 0.6. This finding theoretically supported the substantial positive effect of these social networks on rural residents’ environmental upgrading engagement. In practice, this conclusion was validated: better social and neighborhood relationships among rural residents correlated with improved communication and organizational skills. As a result, environmental protection knowledge circulating within their “relationship circles” was more readily accepted and disseminated [36]. This not only bolstered their own intentions to improve the living environment but also encouraged others within their social networks to participate. The influence of resident-official relationships on environmental upgrading engagement manifested in two key ways: firstly, favorable relationships between residents and officials enhanced residents’ support for the work of village officials, thereby fostering a heightened sense of civic responsibility; secondly, positive relationships facilitated emotional support through communication, making residents more inclined to contribute to collective interests and actively engage in environmental improvement efforts [91]. These findings suggest that a harmonious rural social atmosphere characterized by neighborliness and unity between officials and residents creates a conducive social environment and a solid foundation for improving rural living conditions. Such an environment significantly benefits the enhancement of villagers’ participation in collective actions.

Secondly, in accordance with the Theory of Planned Behavior, subjective norms, perceived behavioral control, and attitudes towards the behavior are key predictors of behavioral intentions. Our empirical research on rural residents’ engagement in environmental upgrading in China supported this framework, aligning with previous studies [17, 18, 20]. We observed that rural residents with stronger subjective norms, higher perceived behavioral control, and more favorable attitudes are more likely to engage actively in efforts to manage their living environment. This conclusion was congruent with the realities in rural China. In rural China, village regulations and customs, which have long been ingrained as value systems, are deeply embedded in residents’ behavioral norms. In some cases, these local regulations may exert a binding force even greater than that of formal legal provisions. When faced with collective action, village regulations and customs can impose significant psychological pressure and perceived loss of benefits on non-participants through mechanisms of public opinion and resource distribution, thereby incentivizing them to join collective efforts [92]. The execution of residential environmental improvement initiatives demands substantial time and effort; thus, the availability of time and labor is a critical and necessary condition for villagers’ engagement in such management [75, 76]. It is undeniable that China is experiencing a moderate-to-severe aging trend, with rural areas aging more rapidly than urban counterparts. Our survey confirmed that a considerable proportion of the individuals residing in rural areas are elderly. For the elderly and those with limited labor capacity, individuals’ engagement in environmental improvement can be challenging, making the support of neighbors particularly vital. Therefore, available convenience is crucial for enhancing villagers’ engagement in living environmental upgrade [77]. Furthermore, we discovered that economically stronger villagers exhibited more positive attitudes towards living environmental improvement. This can be attributed to the fact that individuals with greater economic resources are more likely to prioritize and seek higher-quality living environments, thus demonstrating a greater willingness to invest in environmental enhancements. Finally, the influence of attitudes on behaviors was also confirmed. When villagers perceive environmental improvement as more important, their motivation to actively participate increases, reflecting an evolution in their environmental awareness [61]. This heightened awareness is influenced not only by rational subjective assessments but also by trust in and incorporation of external advice.

Thirdly, the attitudes towards behavior acted as a mediating factor between perceived behavioral control and rural residents’ engagement in improving the living environment, as well as between subjective norms and rural residents’ improving their living environment. Specifically, subjective norms exerted an indirect effect on behavior by shaping individual attitudes towards behavior. As previously noted, individuals living within social groups are subject to the pressures of group norms, prompting them to consciously adjust their behaviors and attitudes to align with these norms [62]. When individuals perceive a strong societal call for and support of environmental improvement, they are more likely to recognize the benefits and necessity of such behavior, leading to the formation of more positive behavioral attitudes and subsequently promoting actual environmental improvement actions. This behavioral logic aligned with cognitive dissonance theory, which posited that when an individual’s behavior and attitudes are inconsistent with group norms, they experience psychological discomfort and are motivated to adjust their behavior and attitudes to alleviate this dissonance [93]. Furthermore, attitudes towards behavior mediated the relationship between perceived behavioral control and rural residents’ engagement in living environment upgrade. Effective environmental management undeniably requires participants to have adequate skills [94], time [76], and economic resources [75]. Consequently, favorable participation conditions and sufficient economic resources are fundamental prerequisites for villagers’ engagement in environmental improvement efforts. When individuals perceive that they can undertake environmental improvement with relative ease (i.e., when they perceive high behavioral control), they are more likely to develop a positive attitude towards these actions. This positive attitude is rooted in their confidence in the successful execution of the behavior, which in turn fosters actual engagement. These findings supported existing research on the relationships between subjective norms, perceived behavioral control, and behavioral attitudes [93], further validating the significant mediating role of attitudes towards behavior.

Fourthly, subjective norms and attitudes towards behavior separately mediated the relationship between social networks and rural residents’ engagement in upgrading the living environment. Specifically, subjective norms mediated this relationship through “soft constraints”. Within social networks, individuals perceive social expectations and norms related to environmental upgrading through interactions and information dissemination. Expectations and support from key figures within the network (e.g., family members, neighbors, or community leaders) are more effectively internalized as subjective norms and self-requirements, thereby motivating engagement. Moreover, environmental actions among neighbors can influence individuals through demonstration effects [9] and peer pressure [10], fostering positive subjective norms and encouraging engagement. The mediating role of attitudes towards behavior played as follows: social networks affect individuals’ attitudes through information transmission, social influence, and behavioral modeling, thereby impacting their behavior. For instance, when network members share positive evaluations or success stories about environmental management, individuals may reassess the value of these actions, forming more positive attitudes. This highlights that behavioral norms and shared values within the network can shape individuals’ attitudes and influence their decisions regarding environmental upgrading.

Finally, employing a chain mediation framework, our study confirmed that social networks exert an indirect influence on rural residents’ engagement in environmental upgrade through the chain mediation effects of subjective norms and attitudes towards behavior. The strength of social networks impacts the intensity of community-imposed norms and pressures, as well as individuals’ attitudes and perceptions toward these norms, thereby influencing their decisions to engage in environmental improvement activities. Specifically, on the one hand, a larger social network provides more opportunities for interpersonal interactions and information exchange, intensifying the influence of subjective norms on individuals’ attitudes and behaviors. On the other hand, subjective norms initially shape individuals’ judgments regarding community expectations and pressures when deciding to engage in environmental improvement. The attitudes formed based on these initial judgments can enhance their positive psychological expectations and offer a more stable prediction of their engagement in environmental management activities. Thus, the chain mediation effects involving subjective norms and behavioral attitudes were confirmed.

## Conclusion

### General conclusion

In the face of escalating global environmental challenges, the efficacy of rural environmental upgrades is pivotal not only for ecological conservation but also for enhancing the quality of life and ensuring social stability among rural populations. In China, rural areas confront pressing environmental issues such as water pollution, soil degradation, and waste management, necessitating immediate and effective interventions [3]. Therefore, a thorough understanding of the mechanisms underpinning rural residents’ engagement in environmental management is essential. Such insights are constructive for advancing environmental protection initiatives and promoting the sustainable development of rural communities.

This study extended the Theory of Planned Behavior by incorporating social network theory, constructing a model that included social networks, subjective norms, perceived behavioral control, and attitudes towards behavior as antecedent variables. The model aimed to elucidate the causal mechanisms underlying rural residents’ engagement in living environment upgrading. Theoretical analysis and empirical testing revealed that social networks and the TPB model variables significantly influenced ELEU. Specifically, social networks impacted ELEU both directly and indirectly through subjective norms and attitudes towards behavior. Additionally, subjective norms and perceived behavioral control influence ELEU indirectly via attitudes towards behavior. Notably, the chain mediation effect of “subjective norms → behavioral attitudes” illustrated the complex influence mechanism of social networks on rural residents’ environmental management behaviors.

### Managerial implications

The findings of this study had the following implications for authorities in developing measures to motivate rural residents to implement living environment upgrading behaviors.

First, local governments should provide the necessary support to foster the development of village social networks. Specifically, it is recommended that government funding be increased for rural public infrastructure projects, including the establishment of senior activity centers and environmental protection associations, which would not only enhance information exchange and resource sharing among residents, but also strengthen community interactions and cooperation. Additionally, Furthermore, mechanisms like anonymous suggestion boxes should be implemented to ensure accessible avenues for villagers to express their diverse environmental concerns and suggestions. Furthermore, village officials should actively seek out and prioritize villagers’ opinions to amplify their influence in local environmental management practices. Moreover, the government should endeavor to create opportunities for more villagers to become key figures within community networks by providing training and support aimed at enhancing their leadership capabilities and organizational skills.

Secondly, as previously noted, one of the primary barriers to rural residents’ engagement in environmental upgrading was their limited understanding of environmental management and a lack of awareness regarding environmental protection. Effectively addressing this challenge necessitates a transformation of conventional propaganda methods and a proactive exploration of optimal information dissemination strategies. Specifically, government agencies and village committees should formulate customized outreach strategies tailored to the diverse needs of various demographic groups, considering factors such as literacy levels, age, and cultural backgrounds. Additionally, a variety of information dissemination methods should be employed—such as organizing environmental protection seminars, distributing educational materials, and leveraging digital media—which not only expands the reach of environmental protection information but also enhances its potential for broader circulation within social networks.

Third, enhancing subjective norms can significantly facilitate rural residents’ engagement in improving their living environments. Consequently, we recommended that village committees conduct regular reviews and revisions of existing village regulations and customs to ensure they are more targeted and practical. Simultaneously, appropriate incentive and penalty measures should be implemented to foster an environment conducive to compliance with these rules. For instance, establishing a reward-and-punishment system for environmental management could encourage active participation while imposing necessary penalties on those who violate the established guidelines. Furthermore, both government entities and village committees should organize regular community activities—such as displaying beautiful courtyard exhibitions—to cultivate positive public awareness and bolster residents’ enthusiasm for environmental management.

Finally, our study further illustrated the exacerbation of population aging and social differentiation trends in rural China. In increasingly hollowed-out areas, the majority of those remaining were middle-aged and elderly individuals. Some villagers exhibited a condition characterized as “willing but not participating” due to personal circumstances. To improve this situation, we proposed the following measures: First, introducing social volunteer services to provide essential support for elderly rural residents with physical limitations. Second, the implementation of public welfare programs for environmental protection skills training to enhance the environmental management proficiency of rural residents. Third, establishing village mutual aid organizations to offer economic and labor assistance to residents in need.

### Limitations and future research

Firstly, the reliance may constrain the generalizability of our research findings on samples solely from Hebei province, given the vast geographical expanse of China and substantial regional differences between the north and south. Future studies could broaden the sample scope to enhance the generalizability of our model. Secondly, the identified influencing factors in this study were limited by the research perspective, and the behavioral decision-making mechanism developed here might only partially explain rural residents’ engagement in living environment upgrade. Therefore, we encourage future research to conduct comprehensive and in-depth investigations into rural residents’ participation behaviors in living environmental upgrade. Thirdly, individual behavioral intentions are shaped by social capital theory (SCT) variables such as social trust, reciprocity, and norms, alongside social networks [95]. In this study, we incorporated social network variables as a supplement to the TPB model, without considering other SCT variables. Hence, we recommend that future research explore further causal relationships between Social Capital Theory and the Theory of Planned Behavior.

## Supporting information

S1 Data (XLSX)

## Abbreviations

AB: Attitudes towards Behavior
ELEU: Rural Residents’ Engagement in Living Environment Upgrade
PBC: Perceived Behavioral Control
SEM: Structural Equation Model
SN: Subjective Norms
SNT: Social Network Theory
SOC: Social Networks
TPB: the Theory of Planned Behavior
